# Adsorption of Pb, Cu, and Ni Ions on Activated Carbon Prepared from Oak Cupules: Kinetics and Thermodynamics Studies

**DOI:** 10.3390/molecules29112489

**Published:** 2024-05-24

**Authors:** Dima Khater, Manal Alkhabbas, Alaa M. Al-Ma’abreh

**Affiliations:** 1 Department of Chemistry, Faculty of Science, Applied Science Private University, Amman 11937, Jordan; 2 Department of Chemistry, Faculty of Science, Isra University, Amman 11622, Jordan; alaa.almaabreh@iu.edu.jo

**Keywords:** adsorption, heavy metal removal, oak-based activated carbon, thermodynamics, kinetics

## Abstract

Agricultural residue-activated carbon and biochar, inexpensive and environmentally friendly adsorbent materials, have recently received significant research attention. This study investigated the potential use of oak cupules in activated carbon form to remove widespread heavy metals (Pb^2+^, Cu^2+^, and Ni^2+^) from wastewater. The oak-activated carbon was prepared from oak cupules and activated with phosphoric acid. Oak-activated carbon was characterized using FTIR, BET analysis, energy-dispersive X-ray spectrometry (EDS), thermogravimetric analysis (TGA), and differential scanning calorimetry (DSC). The Freundlich, Langmuir, and Temkin isotherm models were used to assess the equilibrium data. The impact of various parameters, including pH effect, temperature, adsorbent dose, and contact time, was estimated. The Freundlich model was the most agreeable with Pb^2+^ adsorption by oak-based activated carbon, and Langmuir was more compatible with Cu^2+^ and Ni^2+^. Under optimum conditions, the average maximum removal was 63% Pb^2+^, 60% Cu^2+^, and 54% Ni^2+^ when every ion was alone in the aqueous solution. The removal was enhanced to 98% Pb^2+^, 72% Cu^2+^, and 60% Ni^2+^ when found as a mixture. The thermodynamic model revealed that the adsorption of ions by oak-based activated carbon is endothermic. The pseudo-second-order kinetic best describes the adsorption mechanism in this study; it verifies chemical sorption as the rate-limiting step in adsorption mechanisms. The oak-activated carbon was effective in removing Pb^2+^, Cu^2+^, and Ni^2+^ from wastewater and aqueous solutions.

## 1. Introduction

In many dry countries, treated wastewater has emerged as a potential remedy to alleviate seasonal water shortages brought on by drought. Treated wastewater is crucial for irrigation, industrial uses (cooling, processing), toilet flushing, firefighting, etc. [1]. Local water treatment systems remove organic and heavy metal pollutants using different processes. It is frequently accomplished using challenging and expensive techniques, including ion exchange, membrane filtration [2], and electrochemical deposition [3]. Adsorption and biosorption are among the most efficient methods for removing pollutants, including heavy metals, from wastewater due to their affordability [4], simplicity, safety from secondary contamination [5], effectiveness, and ease of management [6].

The most frequently used adsorbent is activated charcoal (AC). However, AC is expensive because of the processes involved in its regeneration and reactivation [7]. More research has been focused on biochar and agricultural residue-activated carbon because they are inexpensive, renewable, and widely available. Biochar and agricultural residue-activated carbon are carbonaceous substances produced from wood, corn straw, rice husk, pomelo peel, coconut shell, and other agricultural waste [8]. Biochar has textural properties and surface chemical complexity, and it is produced from the thermal decomposition of biomass in the absence or presence of a limited amount of oxygen [9]. Agricultural residue-activated carbon and biochar have been extensively used as adsorbents to remove water pollutants, such as heavy metals [10]. Heavy metals, including lead (II), copper (II), and nickel (II), are among the water supply’s most released and dangerous pollutants and come from industrial processes (e.g., batteries, electroplating, tanneries, textile dyes) and excessive fertilizer use in agriculture [11,12]. These heavy metals are of high concern nowadays. They contribute to bioaccumulation, even at low concentrations, and lead to severe health problems such as cancer and damage to the central nervous system and kidney [13,14].

Many published studies have used agricultural residue-activated carbon and biochar as adsorbents to remove organic and inorganic pollutants, including heavy metals, from water. For instance, Boudrahem et al. [15] developed biochar from coffee residue to remove Cd(II) and Pb(II) from an aqueous solution. Agricultural residue-activated carbon and biochar are more favored by many over synthetic adsorbents, even when their efficiency and selectivity are less than the synthetic ones [16]. They have low production costs, a large surface area, are environmentally friendly, and pave the way for eliminating tons of agricultural residues [17]. Recent improvements in the adsorption capacities of activated carbon have been reported by applying chemical, physical, inorganic, and organic loading strategies [18].

In this study, we prepared and investigated porous oak-based activated carbon as a bio-adsorbent for removing lead (II), copper (II), and nickel (II) from wastewater. Our group previously demonstrated the ability of this adsorbent to remove anionic and cationic dyes [19]. Oak-based activated carbon was derived from oak cupules, an abundant agricultural waste product in northern Jordan. We assessed the impact of various factors, such as solution pH, contact time, temperature, adsorbent dose, and initial metal ion concentration, on the adsorption of metal ions. Our investigation of various adsorption isotherms and kinetic models provides valuable insights into the adsorption process of metal ions.

## 2. Results and Discussion

### 2.1. Characterization of Oak-Activated Carbon

**Figure 1:**
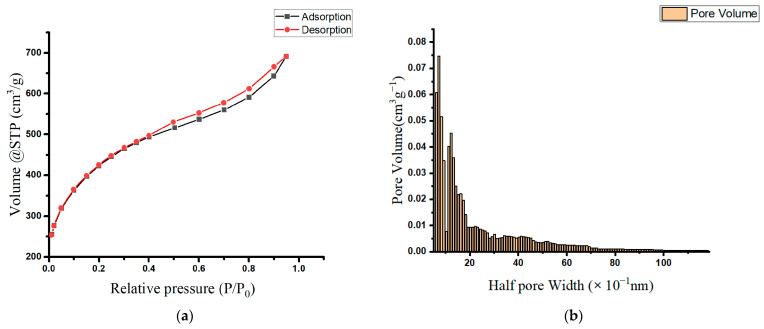
(**a**) Adsorption isotherms of nitrogen at 77 K on oak-activated carbons; (**b**) pore size distribution (PSD) in the oak-activated carbon.

Table 1 lists the prepared oak-activated carbon’s surface area, pore size, and volume measurements. Based on the IUPAC classification, the oak-activated carbon’s N_2_ adsorption/desorption isotherm resembles the type IA isotherm at a relative P/P_0_ less than 0.4, and the type IV and H_3_ hysteresis loops at a relative P/P_0_ greater than 0.4 (Figure 1a). This type of sorption isotherm is characteristic of a combined system of microporous (pore diameter < 2 nm) and mesoporous adsorbents (pore diameter = 2–50 nm) [20,21], demonstrating the presence of a blended structure containing mainly supermicropores and low-range mesopores. Figure 1b presents a histogram representing the extracted pore size distribution (PSD), and it shows that the majority of oak-activated carbon pores falling within the micropore range have a mean pore half width of 0.77 nm (or a pore width of 1.54 nm).

**Figure 2:**
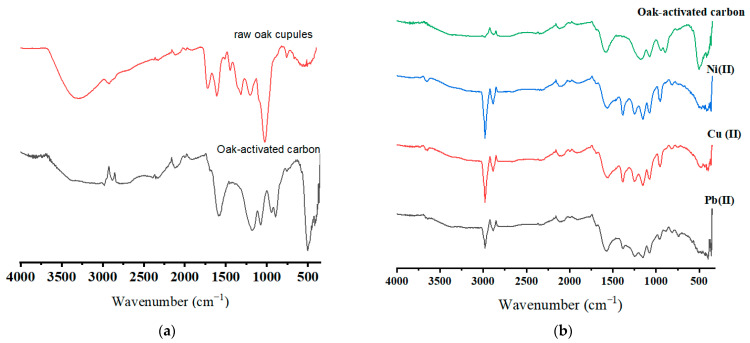
FTIR spectra for (**a**) raw oak cupules and unloaded oak-activated carbon and (**b**) oak-activated carbon loaded with Pb(II), Cu(II), Ni(II), and a heavy metal mixture.

Figure 2a,b display the FTIR spectra of the raw oak cupules and oak-activated carbon (before and after adsorption). A broad band extended from 3200 cm^−1^ to 3650 cm^−1^ is attributed to the hydroxyl group due to lignin and cellulose [22,23]. The hydroxyl group band waned in oak-based activated carbon due to condensation reactions, carbonization, adsorbed water molecule release, and cellulose and hemicellulose decomposition in the raw biomass [24]. The peaks (2924 cm^−1^ in raw oak cupules, 2983 and 2880 cm^−1^ in oak activated carbon) correlate to CH stretching in the CH_2_ and CH_3_ groups. The CH stretching band for the unloaded oak-based activated carbon sample was weak due to the reduced aliphatic compounds after carbonization [25]. The vibration bands with wavenumber 1723 cm^−1^ and 1608 cm^−1^ in the raw oak cupules are related to C=O stretching in unconjugated groups and aromatic C=C stretching vibration in lignin [26]. The small peak at 1200 cm^−1^ shows C-O stretching vibration and may refer to C-O phenol vibration. The IR spectrum reflects various functional groups in raw oak cupules (carbonyls, ester groups, and ketones) [27]. An intensive band in oak-activated carbon appeared at 1587 cm^−1^. This peak may refer to the oxygen–aromatic bonding in the aromatic ether [28], the C=O carbonyl group stretching in the quinone structure, and aromatic C=C vibration [29,30]. The extended absorption band at 1587 cm^−1^ may be attributed to transforming the organic-dominated phases into better-organized aromatic structures. The peak at 1075 cm^−1^ may be caused by ionized linkage (P+ O-) in phosphoric acid [31]. After the adsorption, a slight shift from 1587 cm^−1^ to 1578 cm^−1^ and from 1180 cm^−1^ to 1156 cm^−1^ was observed for all ions. New peaks appeared at 1486 cm^−1^ and 812 cm^−1^ after ions were loaded on oak-activated carbon.

**Figure 3:**
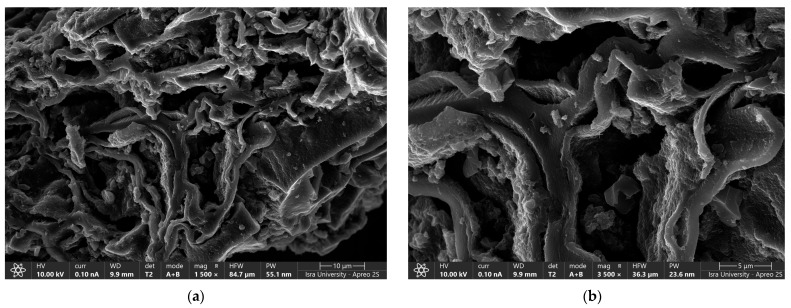
SEM image of oak-activated carbon loaded with a heavy metal mixture.

**Figure 4:**
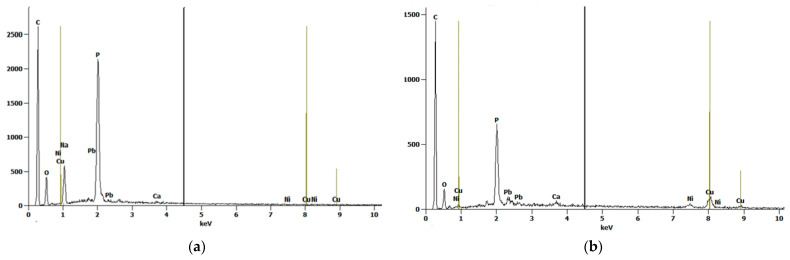
EDS Spectrum for oak-activated carbon (**a**) before adsorption and (**b**) after adsorption.

Figure 3a,b shows the scanning electron microscope (SEM) images of oak-activated carbon after adsorption. The EDS spectrum (Figure 4a) shows that the bulk of oak-activated carbon before adsorption is mostly carbon (61.41% of its mass), oxygen (21.74% of its mass), and phosphorus (11.90% of its mass), with minor amounts of F, Na, Al, Si, and Ca. After the heavy metal mixture sorption, the characteristic peaks of Pb, Cu, and Ni appeared in the EDS spectrum, indicating that heavy metal ions had been successfully adsorbed (Figure 4b).

**Figure 5:**
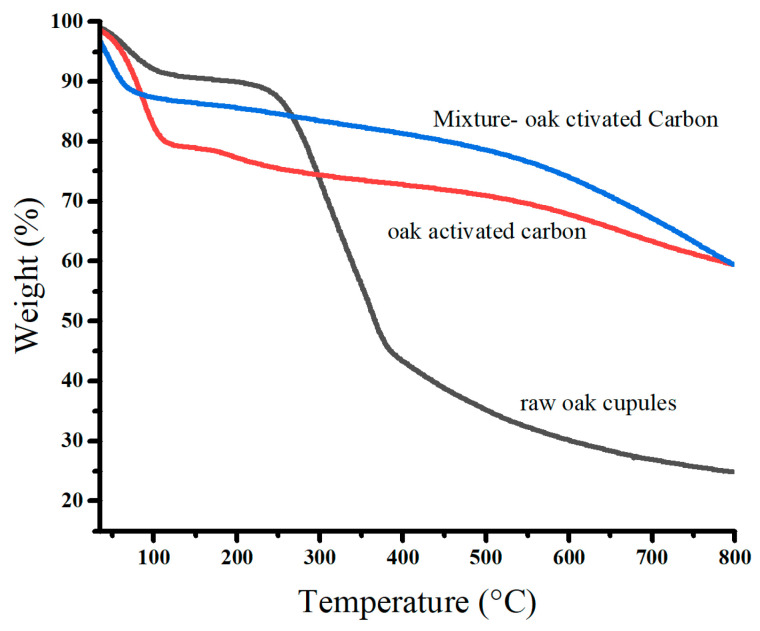
TGA profiles of raw oak cupules and oak-activated carbon.

Figure 5 displays the thermal degradation of raw oak cupules and oak-activated carbon before and after adsorption. We observed that the mass losses occurred in three stages. The first stage of thermal degradation was found at 50–125 °C (dehydration). It has approximate percentage values of the mass losses: 8.95% for raw oak cupules, 20.31% for oak-activated carbon before adsorption, and 11.59% for oak-activated carbon loaded with heavy metals. The water content of oak-activated carbon is at least two times higher than that of raw oak cupules because the developed porosity at the surface of the carbon leads to more agglomerates of water molecules. The water content decreases after biochar adsorbs heavy metal ions, repelling the water molecules occupied by the oak-activated carbon [32].

The second stage of thermal degradation in raw oak cupules (around 300 °C) is associated with the decomposition of hemicellulose, cellulose, and lignin into phenolic compounds and organic acids. This stage leads to a considerable loss of mass (47.45%) [33,34]. However, the second thermal degradation of oak-activated carbon before and after adsorption showed intriguing differences from that of raw oak cupules. The mass loss was significantly smaller (8.24%) and extended from 140 °C to 500 °C, indicating its high thermal stability and low volatile matter and cellulose content [35].

The third thermal degradation stage involves weight losses above 400 °C for raw oak cupules, at which carbonization and graphene sheets grow, and above 500 °C for oak-activated carbon before and after adsorption. It is linked to the breakdown of lignin and the devolatilization of residual char [15,36].

**Figure 6:**
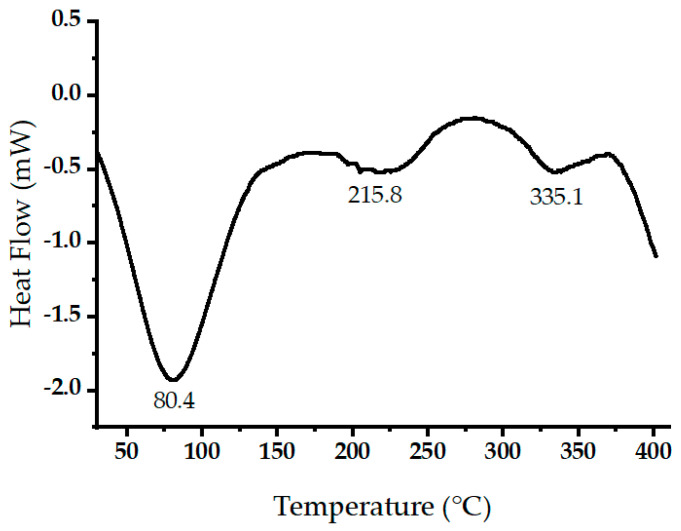
Differential scanning calorimetry (DSC) scans of raw oak cupules.

Figure 6 displays the measured heat flow (in mW) during the thermal decomposition of raw oak cupules within a temperature range of 30 to 400 °C at a heating rate of 10 °C/min. Negative heat flow values indicate endothermic processes. The prominent endothermic peak around 86.3 °C relates to the evaporation of volatile components and water [37]. Two endothermic peaks were detected at 215.8 and 335.05 °C. These two peaks may be due to simultaneous lignin condensation, plasticization, and cellulose thermal decompositions [38,39].

### 2.2. PH Effect

**Figure 7:**
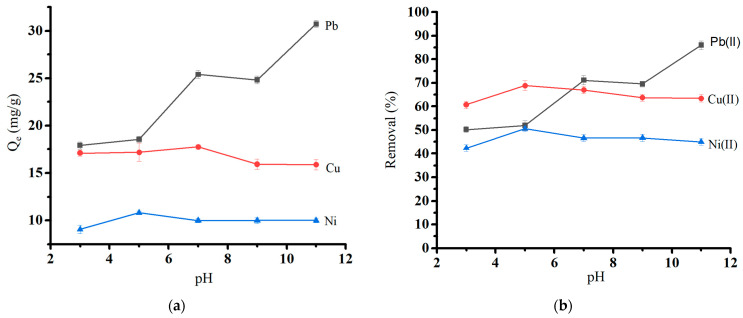
Effect of pH on (**a**) the adsorption capacity of heavy metal ions and (**b**) the removal of heavy metal ions.

The tendency of heavy metal ions to bind to surfaces was investigated at a range of pH values (3.0 to 11.0) using the optimum biochar dosage at initial concentrations of 50 mg/L Pb(II) and 30 mg/L Cu(II) and Ni(II). The pH and biochar’s surface charge distribution influenced the biochar’s heavy metal adsorption. Figure 7a shows the effect of solution pH on the adsorption capacity, and Figure 7b shows the effect of solution pH on the removal (%) of heavy metal ions. The absorption was at a minimum at low pH (pH 3) due to electrostatic repulsion between protonated surfaces and positively charged metal ions (Q_e_ = 17.92 mg/g for Pb(II), 17.11 mg/g for Cu, and 9.07 mg/g for Ni(II)). Sorption capacity gradually increased with rising pH and reached its maximum for Pb (II) at pH 7. At the same time, Cu(II) and Ni(II) showed the most significant sorption capacity at pH 5. Hydronium ions are abundant at low pH and compete with heavy metal ions on active sorption sites [40]. A gradual increase in pH causes an increase in the amount of negative charge on the surface of the biochar, which improves the biochar’s ability to adsorb until pH reaches 7 for Pb(II) and 5 for the other ions. The pH effect is consistent with similar studies involving applying another agricultural waste as an activated carbon source [41,42]. The order of metal removal was Pb(II) > Cu(II) > Ni(II). The additional rise in pH leads to the precipitation of metal hydroxide. This leads to high metal uptake at a PH range of 8 to 11 [43].

Our previous study shows that the pH at zero point charge (pH_pzc_) value for the oak-based activated carbon is around 7.0 ± 0.1 [19]. It is consistent with the pH effect study in the current study. Above pH_pzc_, the adsorbent surface would be negatively charged, and the amounts of heavy metal attached to the adsorbent would increase.

### 2.3. Effect of Adsorbent Dose

**Figure 8:**
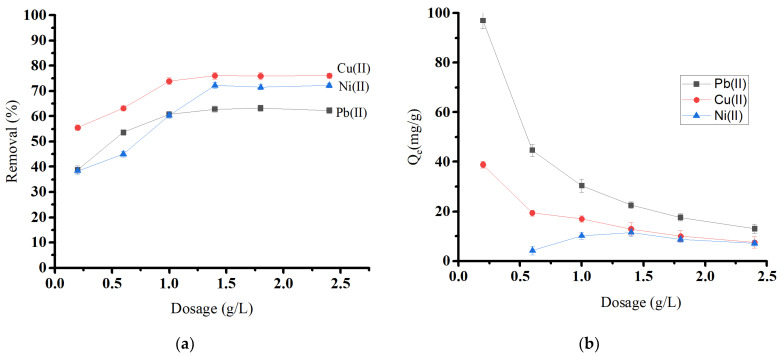
Effect of oak-based activated carbon dosage on (**a**) heavy metal removal and (**b**) heavy metal adsorption capacity.

By equilibrating 0.01 to 0.12 g of biochar material with 50.0 mg/L in 50 mL of a metal solution, the effect of adsorbent dosage on Pb(II), Cu(II), and Ni(II) removal was investigated (Figure 8a). The adsorbent dose is crucial, as it determines the system’s sorbent–sorbate equilibrium. As the amount of adsorbent was increased, the removal percentage increased until it reached equilibrium at a dosage level of 1.4 g/L for all ions. The initial rise in removal percentage comes from the increased biosorbent surface area and the availability of more adsorption sites [44]. After that, the removal percentage became almost constant, which may be attributed to the aggregation, causing a reduction in the overall surface area of the adsorbent [45,46]. Figure 8b shows that increasing oak-based activated carbon dosages decreases the adsorption capacity (Q_e_) for Pb(II) and Cu(II).

### 2.4. Contact Time and Temperature

**Figure 9:**
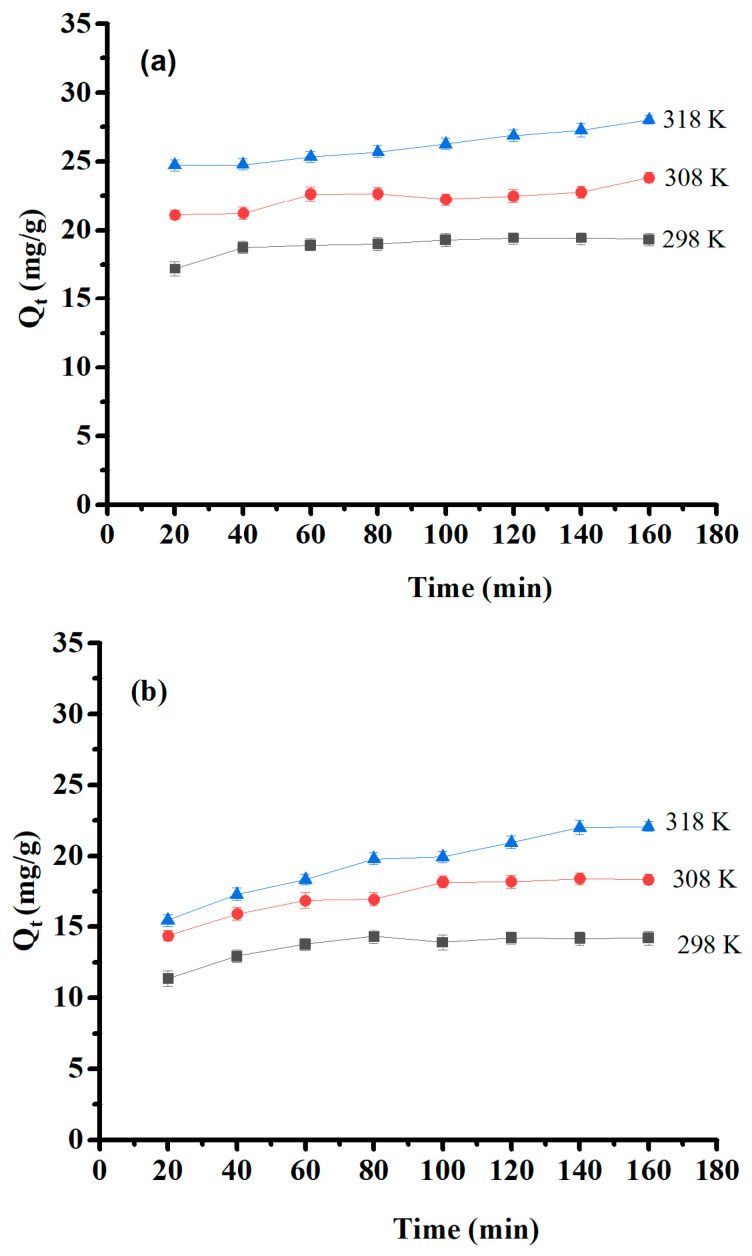

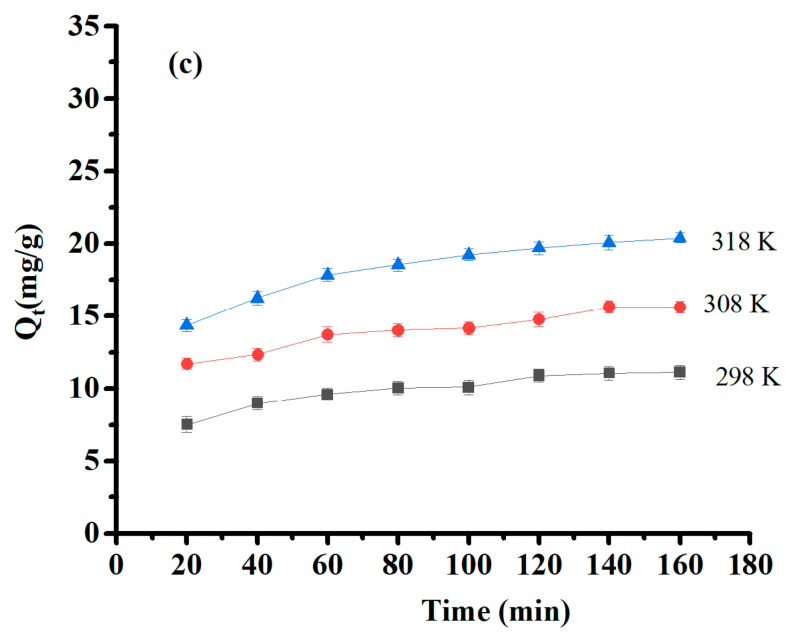
Effect of contact time and temperature on (**a**) Pb(II) adsorption capacity, (**b**) Cu(II) adsorption capacity, and (**c**) Ni(II) adsorption capacity.

The effect of contact time and temperature were estimated within a contact time range of 20–160 min at 25, 35, and 45 °C with initial concentrations of 50 mg/L of Pb (II) and 30 mg/L of Cu(II) and Ni (II). Their results are presented in Figure 9. The optimum time of adsorption was 60 min for Pb(II) and 80 min for Cu(II) and Ni(II). After that, saturation was obtained, and the adsorption capacity remained constant. Furthermore, the experimental results showed that the amount of adsorbed material increased as temperature increased, indicating that the medium’s temperature is a significant factor in adsorption efficiency, and this process is endothermic. The temperature effect is interpreted by increased collision and contact among adsorbates and surface sites on the adsorbent that are available for adsorption [47].

### 2.5. Sorption of Mixed Heavy Metals from Aqueous Phase

**Figure 10:**
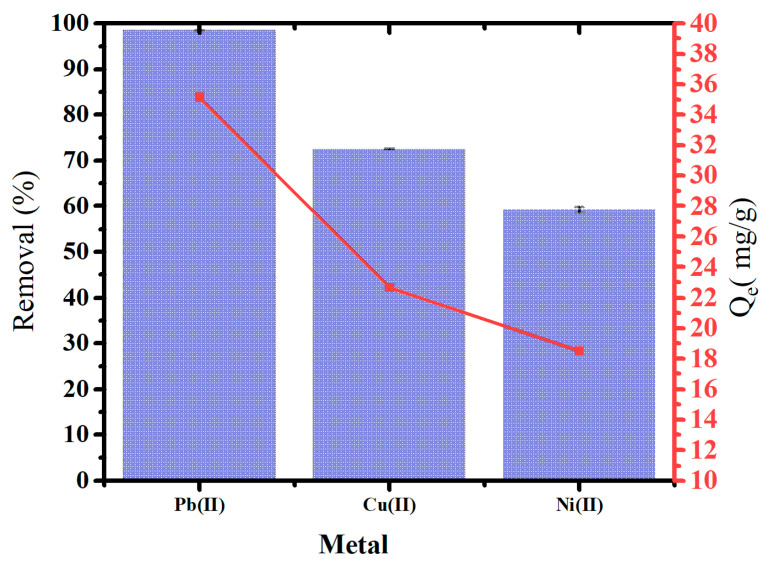
Heavy metal removal and Qe of oak-activated carbon from an aqueous solution containing a mixture of heavy metals.

Figure 10 illustrates the removal of heavy metals and adsorption capacity using oak-activated carbon from an aqueous solution containing mixtures of heavy metals, including Pb(II), Cu(II), and Ni(II). Oak-activated carbon showed a powerful affinity for heavy metal mixtures. The highest removal was for Pb(II) (98.4%), followed by Cu(II) (72.4%) and Ni (II) (60.0%) at 1.4 g/L dosage application. Removing heavy metals from the aqueous phase may involve surface electrostatic interaction, precipitation, and inner and outer surface complexation [48].

### 2.6. Adsorption Isotherms and Kinetics

Analyzing the adsorption isotherm data and comprehending the adsorption mechanisms is crucial. Oak-activated carbon was used to study the behavior of heavy metal adsorption using three isotherm models: Langmuir, Freundlich, and Temkin. The Langmuir model implies monolayer adsorption over an energetically homogeneous adsorbent surface. It disregards interactions between ions that have been adsorbed [49]. Freundlich’s model is based on the adsorbate forming multiple layers on the heterogeneous solid surface of the adsorbent, and the binding strength decreases with increasing site usage [50]. The Temkin model emulates the impact of a few indirect adsorbate/adsorbate interactions. The heat of adsorption would decrease linearly with coverage due to adsorbate/adsorbate interactions [51]. The variables and equations for the chosen isotherm models are described in Section 3.4. Table 2 presents the calculated data based on the Langmuir, Freundlich, and Temkin isotherms. These models were chosen because they comprehensively analyze the adsorption process. Each metal ion was measured individually with 10, 20, 30, 50, and 90 mg/L concentrations at optimum pH. R^2^ values of isotherms are compared to demonstrate how well all isotherms fit the experimental data. The metal ion uptakes on oak-activated carbon experimental data were successfully analyzed. It could be seen that the Freundlich model’s correlation coefficient (R^2^) values were 0.9461 for Pb(II), 0.9476 for Cu(II), and 0.9721 for Ni(II) at 25 °C.

In contrast, Langmuir’s correlation coefficients were 0.9182 for Pb (II), 0.9652, and 0.9934 for Cu(II) and Ni(II), respectively. The Freundlich model was the most appropriate for the adsorption of Pb(II), which means that this ion showed the most adsorption heterogeneity at the bending [52]. Langmuir was the most appropriate for the adsorption of Ni(II), implying the homogeneous distribution of Ni(II) on the active site of the adsorbent [53].

The Langmuir parameter, the dimensionless separation factor (R_L_), is essential in this study, as it is crucial in predicting the affinity between the adsorbate and the adsorbent. R_L_ is computed using the following formula [54,55]:

(1) $$R_{L}=\frac{1}{K_{L}C_{0}+1}$$

where C_0_ represents the adsorbate’s highest initial heavy metal concentration in the solution (mg/L). Depending on the value of R_L_, the isotherm’s shape can be classified as favorable (0 < R_L_ < 1), irreversible (R_L_ = 0), linear (R_L_ = 1), or unfavorable (R_L_ > 1) [56]. The values of R_L_ are offered in Table 2. All R_L_ values are between 0.085 and 0.90, which suggests that heavy metal adsorption onto oak-based activated carbon is highly favorable.

The Freundlich constant (K_F_) is related to adsorption capacity, whereas the constant n deals with the degree of heterogeneity, reflecting the adsorption intensity. n represents favorable chemical adsorption when its value extends from 1 to 10 [57]. In contrast, if n is less than 1, the sorption is considered a physical process. The n range in Table 2 confirms that Pb(II) adsorption is the most favored chemical process.

In the Temkin model, the maximum binding constant (K_T_) value was found for Pb(II), which indicates the most incredible energy for this ion equilibrium adsorption onto the oak-activated carbon.

The correlation between the experimental data and the Langmuir, Freundlich, and Temkin models suggests that metal ion uptakes on oak-activated carbon surfaces are complex and involve multiple simultaneous mechanisms.

**Figure 11:**
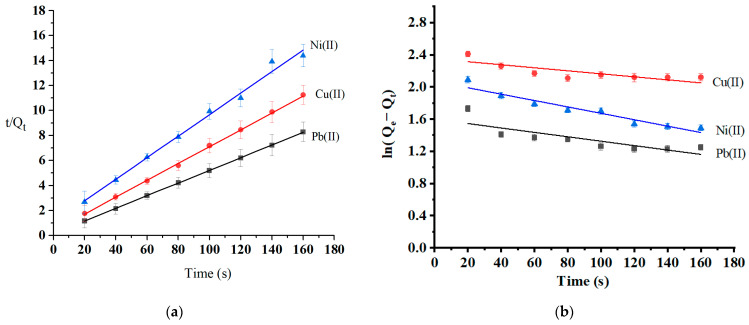
Kinetics models of (**a**) pseudo-second-order and (**b**) pseudo-first-order.

Adsorption kinetics are essential to investigating the adsorption mechanism and the adsorbent’s efficiency. The variables and equations for the selected kinetic studies are reported in Section 3.4. The rate of the adsorption process and the most likely rate-controlling step are well-informed by the adsorption kinetics collected at three different temperatures: 25 °C, 35 °C, and 45 °C. The adsorption kinetics (at optimum pH and initial concentration) were modeled using pseudo-first-order and pseudo-second-order models (Table 3). Figure 11 shows that the adsorption process complies more with the pseudo-second-order kinetic model (R^2^ = 0.9915–0.9999) than the pseudo-first-order model (R^2^ = 0.6273–0.9304). The calculated values of metal ion adsorption capacity from pseudo-second-order kinetics were 19.7 mg/g for Pb(II), 14.7 mg/g Cu(II), and 11.5 mg/g for Ni(II). These values are consistent with experimental values (Qe-experimental (Pb) = 22.9 mg/g, Qe-experimental (Cu) = 14.2 mg/g, and Qe-experimental (Ni) = 11.1 mg/g). The pseudo-second-order model complies with the chemisorption process. The rate-limiting mechanism was the chemisorption of all heavy metal; assuming that chemical interactions such as ion exchange and the chelating reaction caused all heavy metal ions to be adsorbed on the surface of oak-activated carbon [58].

Biochar materials are unique to other materials. Their surfaces can be acidic, basic, hydrophilic, or hydrophobic and can be moved in and out between [59]. Therefore, biochars show complicated mechanisms when interacting with pollutants, including heavy metal ions [60]. This study kept oak-activated carbon at 450 °C for 1 h during pyrolysis. This situation releases some phenolic substances and prevents a fraction of lignin from being biodegraded, causing hydroxyl moieties in polyphenols to bind strongly with metal ions and form a metal–phenolic network (MPN) [61].

The intraparticle diffusion model describes the sorption process and represents a linear plot of Q_t_ versus t^1/2^ [62]. R^2^ and k_id_ are tabulated in Table 3. The predominant controlling step is intraparticle diffusion if this line passes through the coordinate’s origin [63]. All graphs in this study do not pass through the origin point, suggesting that the boundary layers are controlled, and a higher intercept is related to a higher boundary layer’s thickness [64]. Other processes, like adsorption on the external surface (film diffusion), control the rapid initial uptake rate besides the late intraparticle [65]. The Elovich equation describes the second-order kinetics, assuming that the actual solid surfaces are energetically heterogeneous [66]. The value of R^2^ based on Elovich’s model showed linearity with high fitting, suggesting that chemical adsorption may be the mechanism controlling the adsorption process [67]. The α value was the highest for Pb(II) (3.64 × 1013 mg/g min).

### 2.7. Adsorption Thermodynamic Studies

**Figure 12:**
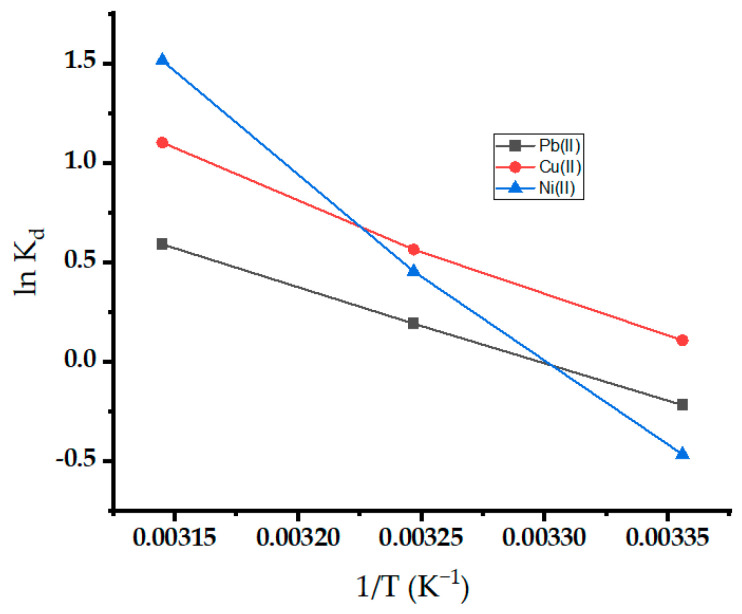
Thermodynamic study for Pb(II), Ni(II), and Cu(II) onto oak-activated carbon.

The thermodynamic parameters for the adsorption of metal ions on oak-activated carbon are estimated in Figure 12. Evaluating the intercept and slope of the plot between lnK_d_ and 1/T yielded the values of ΔS° and ΔH°, respectively [68]. Table 4 lists the thermodynamic parameter values. The negative values of ΔG° at most temperatures demonstrated the viability and spontaneity of the adsorption process, and spontaneity increases with an increase in temperature [69]. The positive value of ∆H° demonstrated the endothermic nature of the adsorption process, and higher temperatures are more favorable for sorption (Figure 12). The observation further proves that the sorbent’s sorption capability increases as temperature rises. The positive value of ∆S° showed increased randomness due to increasing the percentage of active sites at the solution interface as the adsorption progressed [70]. The increased randomness may be attributed to water molecule release at the interface.

### 2.8. Comparison

The performance of oak-activated carbon was compared with that of some synthetic and natural adsorbents derived from agricultural residues. Table 5 shows the results of this comparison. It can be concluded that synthetic adsorbents are more effective than those prepared as agricultural waste-based biochars. Their capacities as adsorbents are higher with faster equilibrium times.

## 3. Materials and Methods

### 3.1. Sample Collection and Preparation of Oak-Based Activated Carbon

The oak cupules were gathered from the Jerash province in Jordan, cleaned of leftover impurities with distilled water, and dried in an air oven at 100 °C for a full day. Then, they were ground and sieved to obtain particle sizes less than 0.5 mm. Oak cupules were mixed with H_3_PO_4_ (85%) at a 3:1 ratio (g H_3_PO_4_/g oak cupules). After being left overnight, the generated slurry was dried at 120 °C for 4 h. The mixture was then cooked for one hour at 450 °C in a muffle furnace. After washing with a 1.0 M NaOH solution to pH 7, distilled water was used to rinse the activated carbon. The prepared oak-based activated carbon was dried at 110 °C for 6 h.

### 3.2. Characterization of Oak-Based Activated Carbon

An Autosorb IQ surface analyzer (Quantachrome, Boynton Beach, FL, USA) was used to investigate textural properties. Nitrogen adsorption and desorption were performed at 77 K. Samples were outgassed at 573 K for 3 h under a 10^−3^ Pa vacuum. The Brunauer–Emmett–Teller equation was used to calculate the BET surface area, whereas the total pore volume V_total_ was estimated at a P/P_0_ relative pressure of 0.99. The nonlocal density functional theory was used to evaluate the pore size distribution (PSD) in the form of slits [77]. Infrared spectra were collected using a Brucker (Billerica, MA, USA) attenuated total reflectance Fourier transform infrared (ATR-FT-IR) spectrophotometer in the 4000–480 cm^−1^ wave number range. The spectra of each sample were recorded at room temperature. An energy-dispersive X-ray spectrometer (EDS) interfaced with the Apreo 2 S LoVac SEM (Thermo Scientific, Waltham, MA, USA) was used to determine the elemental composition of oak-activated carbon before and after adsorption. Thermogravimetric analysis (TGA) was performed using a NETZSCH TG 209F1 thermogravimetric analyzer (Iris, Munich, Germany). A sample of raw oak cupules (7.9 mg), oak-activated carbon before adsorption (11.4 mg), and oak-activated carbon was used to adsorb a mixture of heavy metals (3.1 mg) that had been heated to 110 °C to evolve physically sorbed water. These samples were heated under a nitrogen atmosphere from 25 °C to 800 °C at a heating rate of 10 °C/min. A Shimadzu DSC-60 differential scanning calorimeter (Tokyo, Japan) was used to examine the thermal properties of raw oak cupules. A sealed, empty aluminum pan served as a reference. The sample (3.2 mg) was heated to 110 °C to evolve physically sorbed water. The sample, enclosed in a covered aluminum pan, was heated at 10 °C/min from 30 °C to 400 °C in a nitrogen gas environment.

### 3.3. Adsorption Experiments (Optimum Parameters, Metal Removal Efficiency, and Adsorption Capacities)

Nitrate salts of Cu(NO_3_)_2_, Pb(NO_3_)_2_, and Ni(NO_3_)_2_, all of purity greater than 99%, were dissolved in distilled water to create stock solutions of 1000 mg/L of metal ions that were used in the experiments. Dilutions from the stock solution were applied to have additional concentrations. A calibrated pH meter was used to track the pH changes made to the solutions.

The test solutions were prepared by diluting the stock standard solution with 1000 mg/L Pb(II), Cu(II), and Ni(II) and adding a weighed amount of oak-based activated carbon. The flask’s contents were shaken in an electrically controlled reciprocating shaker for a specified period. After that, they were filtered through a 0.45 µm Millipore filter. Batch-mode adsorption studies of Pb(II), Cu(II), and Ni(II) with oak-activated carbon were conducted in 250 mL stoppered flasks containing 50 mL of the test solution to determine the effect of various parameters, including pH, contact time, adsorbent dosage level, and temperature effect.

Dosage effect was performed at 25 ± 1 °C, agitation of 200 rpm, a contact time of 80 min, and at optimum pH (7 for Pb(II) and 5 for Ni(II) or Cu(II)). The dosage was varied between 0.01 and 0.12 g of biochar material with 50.0 g/L in 50 mL of a metal solution. For the effect of contact time and temperature, the experiments were conducted at 25, 35, and 45 °C, with initial concentrations of 50 mg/L of Pb(II), and 30 mg/L of Cu(II) and Ni(II), agitation of 200 rpm, at optimum pH and optimum dosage.

The heavy metals concentrations were measured using ContraAA800 flame atomic absorption spectrometry (Analytik, Jena, Germany). The following equation determined the percentage of heavy metal ions removed:

(2) $$\mathrm{Removal}\%=\frac{\left(C_{0}{-C}_{e}\right)}{C_{e}}*100\%$$

where C_0_ is the initial metal ion concentration (mg/L) and C_e_ is the equilibrium metal ion concentration (mg/L).

Equilibrium sorption capacity (Q_e_), the amount of metal adsorbed per gram of biosorbent, can be calculated in mg/g using the following equation:

(3) $$Q_{e}=\frac{(\;C_{0}-C_{e})V}{m}$$

where V is the volume of the aqueous solution (L) and m is the adsorbent weight (in g) used.

Equation (4) determines the metal ion adsorption capacity Q_t_ (mg/g) at time t:

(4) $$Q_{t}=\frac{{(C}_{0}-C_{t})V}{m}$$

where C_t_ (mg/L) is the metal ion concentration at time t.

### 3.4. Equilibrium Isotherm and Kinetics Studies

Table 6 lists the variables and equations for the chosen isotherm and kinetic studies models used in this study.

## 4. Conclusions

Simple and inexpensive decontamination processes were applied to remove three widespread and non-biodegradable heavy metals from wastewater, namely, Pb(II), Cu(II), and Ni(II), using oak-activated carbon. Oak-activated carbon proved a promising and easily accessible adsorbent for removing heavy metals from wastewater with low concentrations of Pb(II), Cu(II), and Ni(II), offering a low-cost and renewable method. The effectiveness of oak-based activated carbon in the simultaneous co-adsorption of Pb(II), Cu(II), and Ni(II) was also investigated. Contact times of 60 min for Pb(II) and 80 min for Cu(II) and Ni(II) were enough to achieve the adsorption equilibrium condition. The efficacy of heavy metal removal was the best at oak-active carbon dosage levels of 1.4 g/L. The removal efficiency was the best for Pb(II). A pseudo-second-order model was well adapted to the adsorption kinetics of oak-activated carbon, suggesting heavy metal ions have chemisorption and chelation-directed mechanisms on oak-activated carbon surfaces. Using the pseudo-second-order kinetics model, the calculated Q_e_ agreed well with the experimental Q_e_.

## Figures and Tables

**Table 1:** Textural properties for oak-based activated carbon obtained from N_2_ adsorption at 77 K.

Parameter	Value
BET surface area (m^2^/g)	1088.345
Single point surface area (m^2^/g)	1413.518
Pore diameter (nm)	1.54
Total pore volume (cm^3^/g)	0.985
V_micro_/V_total_	0.724
V_meso_/V_total_	0.196
V_macro_/V_total_	0.065

**Table 2:** Fitting parameters for the Langmuir, Freundlich, and Temkin models.

Parameter	Metal Ion		
	Pb(II)	Cu(II)	Ni(II)
Langmuir			
Q_m_ (mg/g)	7.8	58.5	52.4
K_L_ (L/mg)	0.214	0.054	0.041
R^2^	0.9182	0.9652	0.9934
R_L_	0.085	0.38	0.90
Freundlich			
n	1.9	1.2	1.5
K_F_ (mg/g)	1.2	1.3	1.6
R^2^	0.9461	0.9467	0.9712
Temkin			
K_T_ (L/mg)	1.08	0.22	0.71
b_T_ (J/mol)	1046	241	299
R^2^	0.8438	0.7600	0.9603
B	2.37	10.30	8.28

**Table 3:** Kinetic parameters for the adsorption of Pd(II), Cu(II), and Ni(II) by oak-activated carbon.

Kinetic Model	Parameters	Heavy Metal		
		Pb(II)	Cu(II)	Ni(II)
Pseudo-first order	q_e_, cal (mg/g)	5.0	10.35	8.01
	k_1p_ (min^−1^)	28 × 10^−4^	17 × 10^−4^	41 × 10^−4^
	R^2^	0.6531	0.6273	0.9314
Pseudo-second order	q_e_, cal (mg/g)	19.72	14.71	11.53
	k_2p_ (g/mg min)	1.96 × 10^−2^	1.41 × 10^−2^	7.45 × 10^−3^
	R^2^	0.9999	0.9993	0.9914
Intraparticle	K_id_ (mg/g min^1/2^)	0.4139	0.8228	0.5599
	C	22.3	11.98	11.47
	R^2^	0.9288	0.9864	0.964
Elovich	α (mg/g.min)	3.64 × 10^13^	16.57	18.93
	β (mg/g)	1.90	0.305	0.338
	R^2^	0.9015	0.9798	0.9962

**Table 4:** Thermodynamic parameters for the adsorption of Pd(II), Cu(II), and Ni(II) by oak-activated carbon.

Metal Ion	∆H° (J/mol)	∆S° (J/mol K)	R^2^	∆G° (J/mol)		
				25 °C	35 °C	45 °C
Pb(II)	31,833	105	0.9998	543	−507	−1557
Cu(II)	39,081	132	0.9956	−275	−1575	−2915
Ni(II)	77,804	257	0.9965	1170	−1390	−3961

**Table 5:** Comparison of the adsorption capacity of some synthetic and natural adsorbents for Pb(II), Cu(II), and Ni(II).

Adsorbents	Adsorption Capacities (mg/g)			Equilibrium Time (min)	Adsorbent Materials	Reference
	Pb(II)	Cu(II)	Ni(II)			
Poly-chloromethyl styrene chelating resin	207	167	55	60	Synthetic polymer	[71]
Titanium oxide (TiO_2_) nanofibers	686	835	757	28	Synthetic metal oxide	[72]
Worn tire-activated carbon	-	98	93	40	Synthetic waste	[73]
Hydrous manganese oxide	-	24	30	10	Virgin hydrous manganese oxide	[74]
Modified hydrous manganese oxide	-	31	25	10	Thiol-functionalized hydrous manganese oxide	[74]
Sugarcane-based activated carbon	19	13	3	-	Agricultural waste	[42]
Corn stalk biochar	41	-	-	240	Agricultural waste	[40]
Ragweed and horse weed biochars	124–359	-	-	120	Invasive plant species	[75]
Peanut shell-based biochar	56.5	-	-	180 min	Agricultural waste	[76]

**Table 6:** Equations and parameters of equilibrium isotherms and kinetic models used in this study.

Model Name	Nonlinear Equation	Linear Equation	Parameters	Ref.
Langmuir	$Q_{e}=\frac{K_{L}\mathrm{Ce}}{1+K_{L}\mathrm{Ce}}$Q_m_	$\frac{1}{Q_{e}}=$ $\frac{1}{K_{L}Q_{m}C_{e}}+\frac{1}{Q_{m}}$	Q_m_: Maximum sorption capacity (mg/g). K_L_: Langmuir constant (L/mg).	[49,78]
Freundlich	$Q_{e}=k_{f}C_{e}^{\frac{1}{n}}$	$\ln Q_{e}=\ln k_{f}+\frac{1}{n}\ln C_{e}$	1/n, K_F_: Freundlich constants	[79]
Temkin	$Q_{e}=$$B\;\ln\;(K_{T}C_{e}$) B= RT/b_T_	$Q_{e}=$ $\mathrm{Bln}K_{T}$ $+\mathrm{Bln}C_{e}$	K_T_: Temkin constant related to equilibrium binding energy (L/g). b_T_: Temkin constant related to the sorption heat (J/mol) B: Temkin constant	[80]
Pseudo-first order	$Q_{t}=Q_{e}(1-e^{k_{1p}t}$)	$\ln\left(Q_{e}-Q_{t}\right)=\ln{\mathrm{qQ}}_{e}-k_{1p}t$	k_1p_: Pseudo-first-order rate constant (min^−1^). t: Contact time (min).	[81]
Pseudo-second order	$Q_{t}=\frac{k_{2p}Q_{e}^{2}t}{1+k_{2p\;}Q_{e}}$	$\frac{t}{Q_{t}}=\frac{t}{Q_{e}}+\frac{1}{{k_{2p}Q}_{e}^{2}}$	k_2p_: Rate constant of pseudo-second-order adsorption (g/mg min )	[32]
Intraparticle diffusion	$Q_{t}=k_{\mathrm{int}}t^{\frac{1}{2}}$+ C		$k_{\mathrm{int}}$: Intraparticle diffusion rate constant. C: Intercept	[82]
Elovich	$Q_{t}$ $=\frac{\ln\alpha \beta }{\beta }+\frac{\ln t}{\beta }$		α: Initial adsorption rate (mg/g min). β: Constant related to the extent of surface coverage and activation energy for chemisorptions processes (mg/g).	[83]

## Data Availability

Data are contained within this article.
